# Surgical Management of Irreparable Rotator Cuff Tears: A Survey of Current Practice and Literature Review

**DOI:** 10.7759/cureus.67907

**Published:** 2024-08-27

**Authors:** Akhilesh Pradhan, Kapil Kumar, Alexandra Haddon, Peter Brownson, Harvinder Singh

**Affiliations:** 1 Trauma and Orthopaedics, University Hospitals of Leicester NHS Trust, Leicester, GBR; 2 Trauma and Orthopaedics, Grampian Orthopaedic and Trauma Unit, Woodend General Hospital, NHS Grampian, Aberdeen, GBR; 3 Trauma and Orthopaedics, Liverpool Upper Limb Unit, Liverpool University Teaching Hospitals NHS Foundation Trust, Liverpool, GBR

**Keywords:** shoulder pseudoparalysis, shoulder girdle, shoulder injuries, orthopaedic trauma surgery, rotator cuff tears

## Abstract

Introduction

Irreparable rotator cuff tears (RCTs) are a complex challenge encountered by shoulder surgeons. Despite a range of repair strategies, the preferences and indications of these remain unclear. Our study aims to identify current practices, preference for graft choice and indications for capsular reconstruction amongst UK-based surgeons.

Methods

An online survey was sent to members of the British Elbow and Shoulder Society (BESS). Procedural preferences, operative frequency, indications/contra-indications for superior capsular reconstruction (SCR) and graft choice were ascertained. An independent t-test was used to determine statistical significance.

Results

One hundred and ten upper limb surgeons responded to the survey. Of this cohort, 90/110 (81.8%) would be able to perform a partial cuff repair, 89/110 (80.9%) could offer a reverse shoulder arthroplasty (RSA) and 82/110 (74.6%) could perform debridement only. Less commonly, 35/110 (31.8%) could offer an Inspace^TM^ balloon device, 31/110 (28.2%) SCR and 16/110 (14.6%) a tendon transfer. None of the respondents had performed more than 10 Inspace^TM^ balloons in the previous year. 72/105 (68.6%) had never performed a SCR and 86/105 (82%) had never performed a tendon transfer. Over 58/105 (55.2%) had performed >10 RSA in the previous year. The graft of choice for SCR was human dermal allograft 33/100 (33%) and this choice was most frequently guided by surgeon preference.

Conclusion

Our study demonstrates that various treatment options can be offered for the management of irreparable RCTs. The commonest procedure offered is a partial cuff repair followed by RSA. Newer, novel procedures such as Inspace^TM^ balloon and tendon transfers are less commonly offered in UK-based practices and their indications for use are less well defined. Future high-powered, multi-centre studies are required to identify the role and outcomes of these procedures.

## Introduction

Rotator cuff tears (RCTs) are a common cause of shoulder pain [1,2]. The incidence of cuff tears increases with age and over 65% of those >70 years have either an incidental or symptomatic cuff tear. Surgical treatment is indicated for patients who remain symptomatic despite non-operative management [3-6]. An RCT is irreparable if it is not possible to achieve direct fixation of the torn tendon to its insertion site. This may be due to the size of the tear, retraction or muscle impairment caused by atrophy and fatty infiltration, resulting in poor healing potential [6-9]. The term irreparable is often used interchangeably with massive; but whilst most irreparable RCTs can also be considered massive tears, not all massive tears are irreparable [10]. The management of irreparable RCTs ranges from non-operative management such as anterior deltoid strengthening physiotherapy or steroid injections to operative options including debridement, partial cuff repair, augmentation (either open or arthroscopic), InSpace^TM^ balloon, superior capsular reconstruction (SCR), tendon transfers and reverse shoulder arthroplasty [6,7,11-17].

The purpose of our study was to gauge the current practice, amongst British Elbow and Shoulder Society (BESS) members, for the surgical management of irreparable RCTs. We wished to assess the procedures offered, frequency of intervention, and choice of grafts and to determine what factors influence their decisions.

## Materials and methods

An online questionnaire was sent to all surgeon members of the BSS relating to the management of irreparable RCTs. This was created using the online tool SurveyMonkey (http://www.surveymonkey.com). Approval for the study was obtained from the BESS Research Committee. Research ethics committee approval was not sought for this surgeon-only, non-patient-facing survey. The study period was inclusive of 06/05/2020-07/07/2020.

The survey used quantitative questioning to assess the preferred procedures used in the treatment of irreparable RCTs, with the example shown in Table 1. For reference, the full questionnaire is included in the appendix (table in the Appendix). The questionnaire delineated procedural familiarity, choice of SCR graft, reasoning for graft choice and absolute and relative contraindications for performing SCR. The survey also included clinical experience (surgeon grade at the time of completion). An independent t-test was used to identify if there was statistical significance (p<0.05). 

**Table 1:** Overview of the Quantitative Questions Used to Assess the Preferred Procedures in the Management of Irreparable RCTs RCTs: Rotator cuff tears

	Do You Perform This Procedure for Irreparable RCTs?	How Many Do You Perform Per Year?			
Debridement Only	Yes / No	None	0-5	5-10	>10
Partial Cuff Repair	Yes / No	None	0-5	5-10	>10
Augmentation (Open or Arthroscopic)	Yes / No	None	0-5	5-10	>10
InSpace^TM^ Balloon	Yes / No	None	0-5	5-10	>10
Superior Capsular Reconstruction	Yes / No	None	0-5	5-10	>10
Tendon Transfers	Yes / No	None	0-5	5-10	>10
Reverse Shoulder Arthroplasty	Yes / No	None	0-5	5-10	>10

## Results

One hundred and ten specialist upper limb surgeons responded to the survey. Of these, 106/110 (96%) were consultant surgeons, 2/110 (2%) were fellows and 2/110 (2%) were senior trainees (ST7-8). Not all surgeons completed each individual question and therefore percentages are calculated as per the question response rate.

Surgical intervention

A wide range of surgical options were presented to respondents regarding treatment offered to patients with irreparable RCTs. The most commonly offered procedures were partial cuff repairs and RSA. 90/110 (81.8%) would be confident in offering a partial cuff repair whilst 89/110 (80.9%) would offer RSA. There was no statistical significance in participants preferring either treatment (p=0.50). 82/110 (74.6%) of surgeons would consider debridement alone. 35/110 (31.8%) would perform an InSpace^TM^ balloon, 31/110 (28.2%) SCR and 16/110 (14.55%) would perform a tendon transfer (Table 2). There was no statistical significance between respondents choosing SCR and tendon transfer (p=0.98) and similarly there was no statistically significant difference between surgeons offering partial cuff repair versus SCR (p=0.50). 

**Table 2:** Procedures Performed in the Management of Irreparable RCTs Comparison of SCR between the different treatment modalities is used to calculate p-values and statistical significance. * denotes statistical significance. RCTs: Rotator cuff tears; SCR: superior capsular reconstruction

Procedure	N (%)	p-value*
Debridement Alone	82/110 (74.55%)	p=0.60
Partial Cuff Repair	90/110 (81.82%)	p=0.54
Augmentation (Open or Arthroscopic)	50/110 (45.45%)	p=0.86
InSpace^TM^ Balloon	35/110 (31.82%)	p=0.97
Superior Capsular Reconstruction	31/110 (28.18%)	
Tendon Transfers	16/110 (14.55%)	p=0.91
Reverse Shoulder Arthroplasty	89/110 (80.91%)	p=0.55
Other	9/110 (8.18%)	p=0.87

Frequency of procedural intervention

The frequency of the most commonly and least commonly offered procedures was assessed. 98/105 (93.3%) of surgeons had performed at least one RSA procedure in the previous year. 17/105 (16.2%) had performed between 5 and 10 RSA procedures whilst 58/105 (55.2%) had performed >10 RSAs in the previous year (Table 3). There was no statistically significant difference between surgeons performing at least one RSA versus surgeons performing >10 RSA procedures (p=0.41). 66/106 (62.3%) of surgeons had not performed a single InSpace^TM^ balloon whilst only 33/106 (31.3%) had performed up to five procedures in the previous year with no statistical significance (p=0.72). 86/105 (81.9%) had not performed a single tendon transfer in the previous year. 16/105 (15.2%) had performed up to five tendon transfers in the previous year; there was no statistically significant difference (p=0.52). Only 1/105 (1.0%) had performed >10 tendon transfers in the previous year. 72/105 (68.6%) had not performed a single SCR in the previous year whilst only 2/105 (1.9%) had performed up to 10 SCR procedures in the previous year and a similar 2/105 (1.9%) had performed >10 procedures in the previous year, not statistically significant (p=0.58). 

**Table 3:** Number of Procedures Performed for Irreparable RCTs Per Year p-values demonstrating significance between surgeons performing zero cases and between 5 and 10 procedures, * denotes statistical significance. RCTs: Rotator cuff tears

Procedure	None	1-5 procedures	5-10 procedures	>10 procedures	p-value*
InSpace^TM^ Balloon	66/106 (62.26%)	33/106 (31.13%)	7/106 (6.6%)	0	p=0.63
Superior Capsular Reconstruction	72/105 (68.57%)	29/105 (27.62%)	2/105 (1.9%)	2/105 (1.9%)	p=0.58
Tendon Transfers	86/105 (81.90%)	16/105 (15.25%)	2/105 (1.9%)	1/105 (0.95%)	p=0.50
Reverse Shoulder Arthroplasty	7/105 (6.67%)	23/105 (21.90%)	17/105 (16.19%)	58/105 (55.24%)	p=0.94

Superior capsular reconstruction

Surgeons were further questioned regarding SCR, the most common graft choice, decision-making processes, and perceived absolute and relative contraindications to SCR. 33/100 (33%) of surgeons would opt for a human dermal allograft, the most common graft choice. 9/100 (9%) would prefer a synthetic patch whilst 1/100 (1%) would use fascia lata (FL) graft. There was no statistically significant difference between surgeons choosing human dermal allograft and FL graft (p=0.81). Most respondents would not perform SCR and therefore did not have a graft preference. Reasoning for graft choice was most commonly based upon surgeon preference 47/100 (47%), whilst 18/100 (18%) felt hospital management/their Trust influenced graft choice/availability.

Regarding perceived absolute and relative contraindications to SCR, the most common absolute contraindication for SCR was grade 3 Kellgren-Lawrence degenerative change, 82/100 (82%). The second most common absolute contraindication stated was an irreparable tear of subscapularis, 68/100 (68%) followed by the absence of infraspinatus tendon 48/100 (48%). The least common absolute contraindication for SCR was the presence of a reparable tear of subscapularis, 8/100 (8%), followed by a previously failed rotator cuff repair, 9/100 (9%), and active forward flexion <90 degrees on clinical examination, 10/100 (10%). Regarding relative contraindications, respondents felt that superior migration of the humeral head 38/98 (38.8%) was the most common factor. This was followed by a reparable tear of subscapularis 32/98 (32.7%) and the absence of infraspinatus tendon 30/98 (30.6%). A previously failed rotator cuff repair was the least reported relative contraindication (Table 4).

**Table 4:** Perceived Relative and Absolute Contraindications to Performing SCR p-value demonstrating statistical significance between absolute and relative contraindications, * denotes statistical significance.

Contraindication	Relative (n=98)	Absolute (n=100)	p-value*
Superior Migration of the Humeral Head (%)	38/98 (38.78%)	33/100 (33%)	p=0.97
Grade 3 Degenerative Changes (%)	27/98 (27.55%)	82/100 (82%)	p=0.51
Reparable Tear of Subscapularis (%)	32/98 (32.65%)	8/100 (8%)	p=0.86
Irreparable Tear of Subscapularis (%)	22/98 (22.45%)	68/100 (68%)	p=0.61
No Infraspinatus Tendon (%)	30/98 (30.61%)	48/100 (48%)	p=0.83
Previous Failed Rotator Cuff Repair (%)	18/98 (18.37%)	9/100 (9%)	p=0.95
Active Forward Elevation <30^o ^(%)	29/98 (29.59%)	28/100 (28%)	p=0.99
Active Forward Elevation <90^o ^(%)	22/98 (22.45%)	10/100 (10%)	p=0.94

## Discussion

The surgical management of irreparable RCTs remains a complex and multifactorial issue. Our study demonstrates that there is no clear consensus on the operative management of these injuries and several procedures are considered as common methods of repair/reconstruction. Our results reflect the challenges faced in tailoring management options toward the correct indication in individual patients which have been explored in previous literature reviews. Often, more than one procedure may be indicated to augment the repair strategy [6,11,12,14,15]. As an example, arthroscopic debridement may assist in pain management but does not provide any significant improvement in strength [18]. In contrast, biceps tenotomy, partial rotator cuff repair and suprascapular nerve release improve both pain and strength [6,10]. At the end of the treatment spectrum, for low-demand elderly patients, RSA provides a beneficial option to improve the range of motion and strength and address pain management issues [12].

Current evidence reports that despite advances in the surgical management of irreparable RCTs, there is no current gold standard or consensus on the most superior operative intervention. The importance of tailoring treatment to patient requirements and demographics is key to successful management of these patients; SCR has demonstrated excellent outcomes in some patients but remains less successful in patients with irreparable subscapularis tears [13]. A recent systematic review identified that the definition of ‘irreparable’ varies greatly between studies and populations and that variability in patient demographics and length of follow-up renders direct comparison of treatment options difficult. Overall, positive clinically important effects were reported for all surgical treatment modalities [19].

Whilst procedures such as partial rotator cuff repair and RSA are well established, as reflected in our results, the relative number of more novel, targeted procedures is small, especially the use of InSpace^TM^ balloon, SCR and tendon transfers which our results demonstrate have a surgical frequency of typically <5 cases per year. Whilst the literature demonstrates the clinical utility of partial cuff repair in younger, less co-morbid patients and RSA in older, less active patient demographics, our study did not explore the specific indications for each procedure [20]. Furthermore, the small numbers of SCR and tendon transfers being performed indicate the need for further research to identify the specific indications and the long-term outcomes for each treatment option. The literature demonstrates that SCR may improve short-term patient-reported outcome measures and pain scores but there remains a paucity of data supporting long-term outcomes, with a varying rate of failure rates documented between studies [20].

The SCR procedure was initially described in 2013 and aimed to restore the superior stability of the shoulder joint. The original technique described the use of a thick FL allograft patch with medial anchoring to the superior glenoid and laterally at the cuff footprint on the greater tuberosity, then suturing the graft to the infraspinatus tendon and remaining supraspinatus or subscapularis tendon [21]. Subsequently, newer variations of this technique were introduced e.g., the use of dermal allograft, popularised in the United States [22,23].

A biomechanical study comparing the ability of FL to restore superior stability to human dermal allograft demonstrated that SCR using FL allograft completely restored superior translation, subacromial contact pressure and superior glenohumeral joint force, whereas human dermal allograft only partially restored superior glenohumeral stability [24].

Subsequent literature has suggested that graft thickness may be important. In his initial description, Mihata used 9mm thick FL grafts, whereas dermal allografts tend to be 3-4mm thick, although a systematic review comparing FL and dermal graft thickness concluded that both are acceptable [25-28].

Our study demonstrated that only 1/100 (1%) of respondents would use an FL graft, whereas 33/100 (33%) would use human dermal allograft. 47/100 (47%) attributed their graft choice to personal decisions, and 18/100 (18%) felt that it was influenced by their trust/hospital policies. With the current literature implying that FL graft provides superior outcomes to dermal allograft unless double thickness is used, concerns are raised about whether this variation may affect surgeon’s perception of the procedure and its outcomes. Future studies could assess the thickness of grafts used by surgeons and whether ‘double thickness’ grafts were administered in certain units.

The original paper by Mihata et al. (2013) did not specify any contraindications; however, the exclusion criteria included severe bone deformity, severe superior migration of the humeral head that did not move with the traction of the arm, cervical nerve palsy, axillary nerve palsy, deltoid muscle dysfunction and infection [21]. 

Our results demonstrated discrepancies between original exclusions by Mihata et al. and the current practice among the BESS surgeon members. Whilst 33/100 (33%) felt that superior migration of the humeral head was an absolute contraindication, 38/98 (38.78%) felt it was only a relative contraindication. In agreement with the original paper, 82/100 (82%) felt that grade 3 glenohumeral osteoarthritis was an absolute contraindication to a SCR. 

Mihata et al. did not comment on the presence of a viable infraspinatus in their original paper; however, a subsequent cohort study concluded that a subscapularis tear affected clinical outcomes and resulted in reduced strength and range of movement of the shoulder; there was no difference in post-operative outcomes for intact or reparable subscapularis tear [13,29]. Our results reflected this finding, with 68/100 (68%) of respondents stating that an irreparable subscapularis tear was an absolute contraindication to SCR. However, 32/98 (32.65%) felt that a reparable tear was a relative contraindication contradicting the evidence stated in the literature.

Burkhart et al. demonstrated pseudoparalysis of the shoulder (active forward elevation less than 45^o^) was reversed/markedly improved in 90% of patients after SCR [30]. Similarly, Mihata et al. observed that 93% of patients with severe pseudoparalysis of the shoulder (active forward elevation less than 90^o^ with positive drop arm sign) demonstrated significant improvement in their pseudoparalysis post-SCR [29]. Our results are not directly comparable to these findings, but only 10/100 (10%) reported active forward elevation <90^o^ was an absolute contraindication with 22/98 (22.5%) stating this as a relative contraindication for SCR. Only 28/100 (28%) reported that active forward elevation less than 30^o^ was an absolute contraindication. 

The biggest limitation of our study is the survey format; this is a low-level form of evidence and therefore limits the conclusions that can be drawn from the results. Further limitations include the low overall response rate and selection bias towards surgeons more likely to respond to voluntary internet-based questionnaires, hence, limiting the ability to extrapolate these results to the entire cohort of UK-based shoulder surgeons. The questionnaire was composed of quantitative questions, but a longer period for questionnaire completion may have led to increased responses, adding further weight to the findings. To encourage questionnaire completion, only six key questions were posed. Upon review of the available literature, further information may have been obtained by expanding the question base to include the perceived definition of irreparable RCT as well as demographic and clinical considerations for conducting specific interventions.

Overall, our study provides a useful insight into current practices for the management of irreparable RCTs, specifically highlighting that there is no firm consensus on which operative intervention is considered the gold standard. Similarly, it highlights the perceived disagreement between surgeons regarding which factors constitute relative versus absolute contraindications for performing the SCR procedure. Whilst current evidence supports positive short-term post-operative outcomes for all surgical interventions, it is evident that further research is required using high-powered, multi-centre trials to determine an operative gold standard regarding both clinical outcomes and cost efficiency.

## Conclusions

Our findings demonstrate the use of multiple different procedures in the management of irreparable RCTs amongst UK-based surgeons. The management of irreparable RCTs is complex and often involves multi-factorial decision-making processes. Whilst the use of RSA in older, less active patients and partial rotator cuff repair in younger, active patients is widely accepted, the place of other procedures is less clearly defined. There remains equipoise in offering patients procedures such as InSpace balloons, tendon transfers and SCR. Most of the survey participants did not perform >5 SCR procedures, InSpace balloons or tendon transfers. There was no consensus on the graft choice and the most cited reason for a particular graft type was surgeon preference. Furthermore, there was variance in reasons listed as absolute and/or relative contraindications for the SCR procedure; however, most participants accepted grade 3 degenerative changes to be an absolute contraindication for the SCR procedure. Ultimately, further multi-centre studies are required to identify the role and long-term clinical outcomes of these procedures.

## Appendices

**Table 5:** Full Questionnaire Sent to BESS Participants for Completion of the Survey RCTs: Rotator cuff tears; SCR: superior capsular reconstruction; BESS: British Elbow and Shoulder Society

	Do You Perform This Procedure for Irreparable RCTs?	How Many Do You Perform Per Year?			
Debridement Only	Yes / No	None	0-5	5-10	>10
Partial Cuff Repair	Yes / No	None	0-5	5-10	>10
Augmentation (Open or Arthroscopic)	Yes / No	None	0-5	5-10	>10
InSpace^TM^ Balloon	Yes / No	None	0-5	5-10	>10
Superior Capsular Reconstruction	Yes / No	None	0-5	5-10	>10
Tendon Transfers	Yes / No	None	0-5	5-10	>10
Reverse Shoulder Arthroplasty	Yes / No	None	0-5	5-10	>10
	Which Graft Do You Use for SCR?	How Many Do You Perform Per Year?			
Fascia Lata Graft	Yes / No	None	0-5	5-10	>10
Human Dermal Allograft	Yes / No	None	0-5	5-10	>10
Synthetic Patch Graft	Yes / No	None	0-5	5-10	>10
	What is Your Choice of Graft Based on?				
Influence by Trust/Hospital Management	Yes / No				
Personal Preference	Yes / No				
	Which Do You Consider an Absolute Contraindication to Performing SCR?				
Superior Migration of Humeral Head	Yes				
Grade 3 Degenerative Changes	Yes				
Reparable Tear of Subscapularis	Yes				
Irreparable Tear of Subscapularis	Yes				
Previous Failed Rotator Cuff Repair	Yes				
Active Forward Elevation <30 degrees	Yes				
Active Forward Elevation <90 degrees	Yes				
	Which Do You Consider a Relative Contraindication to Performing SCR?				
Superior Migration of Humeral Head	Yes				
Grade 3 Degenerative Changes	Yes				
Reparable Tear of Subscapularis	Yes				
Irreparable Tear of Subscapularis	Yes				
Previous Failed Rotator Cuff Repair	Yes				
Active Forward Elevation <30 degrees	Yes				
Active Forward Elevation <90 degrees	Yes				
	What Is Your Grade?				
Senior Trainee (ST7/8)	Yes / No				
Fellow	Yes / No				
Consultant	Yes / No				
